# Nepal Introduces Free Thrombolytic Drugs for Acute Ischemic Stroke: A Revolutionary Step in Acute Stroke Care

**DOI:** 10.31729/jnma.8958

**Published:** 2025-04-30

**Authors:** Bibek Rajbhandari, Bikram P Gajurel, Bikash Devkota

**Affiliations:** 1 Maharajgunj Medical Campus, Tribhuvan University Institute of Medicine, Maharajgunj, Kathmandu, Nepal; 2 Ministry or Health and Population (MoHP), Nepal

## INTRODUCTION

Nepal is set to provide free thrombolytic drugs, Alteplase Recombinant, to treat acute ischemic stroke, a major health concern in the country.^1,2^ Stroke accounts for 7.6% of total deaths and 3.5% of the total disability-adjusted life years (DALYs) in Nepal.^3^ This initiative, supported by Direct Relief, aims to reduce the burden of stroke in the country, with the medicine being made available at key tertiary care hospitals, including Bir Hospital, Tribhuvan University Teaching Hospital, and Bharatpur Hospital.^1^ Further, Ministry of Health and Population (MoHP) is planning to expand this initiative to other tertiary centers, such as Patan Hospital, based on the burden of acute ischemic stroke and its management.

The global treatment landscape for stroke has undergone a revolutionary shift, particularly with the advent of thrombolysis. The timely administration of thrombolytic therapy within a specified window period can significantly improve outcomes for acute ischemic stroke patients.^4^ Thrombolysis, primarily using Alteplase, has become a cornerstone of stroke treatment worldwide, providing the potential to save lives and reduce long-term disability. However, despite its proven effectiveness, thrombolytic drugs remain prohibitively expensive for patients in low- and middle-income countries (LMICs). In these regions, the cost of Alteplase, which can range from $650 to $1,000 per 50-mg vial, places a significant financial burden on patients and healthcare systems.^5^

Our study conducted at the Teaching Hospital (TUTH) revealed that out of 1,177 stroke cases identified over the course of a year, 76% were ischemic strokes.^6^ However, only 22 patients (1.9%) received thrombolysis. Financial barriers were identified as a major reason for the delay in treatment. The high cost of thrombolytic drugs, such as Alteplase, often made them unaffordable for many patients, preventing timely administration of the treatment within the critical window period.^6^ This data, which comes from just a tertiary care center with significant case records, highlights the need for broader attention. It is essential to consider the burden in other regions and among those who did not receive treatment, for whom we do not have data. Therefore, when we look at the larger picture, this situation represents just the tip of the iceberg.

This initiative by the Nepalese government marks a major milestone in stroke care in the country. Providing free access to Alteplase will not only support patients who would otherwise be unable to afford this life-saving treatment, but it will also contribute to an increase in the quality of life for stroke survivors. Additionally, it will reduce the long-term disabilities that often result from untreated strokes.

While this step is crucial, there are still several challenges to address. The government must also focus on increasing awareness about stroke symptoms and the importance of seeking timely medical care, as these are major barriers contributing to delays in treatment. The availability of thrombolytic drugs in rural areas, where access to healthcare services is often limited, must also be prioritized, as the window period for effective thrombolysis is critical.

This initiative represents a significant advancement in stroke care in Nepal, but it is just the beginning. Continued efforts to improve access to treatment, enhance awareness, and strengthen healthcare infrastructure are necessary to ensure that all stroke patients can benefit from this life-saving therapy.
